# Comparison of Gait Speed Reserve, Usual Gait Speed, and Maximum Gait Speed of Adults Aged 50+ in Ireland Using Explainable Machine Learning

**DOI:** 10.3389/fnetp.2021.754477

**Published:** 2021-11-05

**Authors:** James R. C Davis, Silvin P. Knight, Orna A. Donoghue, Belinda Hernández, Rossella Rizzo, Rose Anne Kenny, Roman Romero-Ortuno

**Affiliations:** ^1^ The Irish Longitudinal Study on Ageing, Trinity College Dublin, Dublin, Ireland; ^2^ Discipline of Medical Gerontology, School of Medicine, Trinity College Dublin, Dublin, Ireland; ^3^ Mercer’s Institute for Successful Ageing, St James’s Hospital, Dublin, Ireland

**Keywords:** gait, speed, maximum, reserve, explainable, gradient, walking, physiological

## Abstract

Gait speed is a measure of general fitness. Changing from usual (UGS) to maximum (MGS) gait speed requires coordinated action of many body systems. Gait speed reserve (GSR) is defined as MGS–UGS. From a shortlist of 88 features across five categories including sociodemographic, cognitive, and physiological, we aimed to find and compare the sets of predictors that best describe UGS, MGS, and GSR. For this, we leveraged data from 3,925 adults aged 50+ from Wave 3 of The Irish Longitudinal Study on Ageing (TILDA). Features were selected by a histogram gradient boosting regression-based stepwise feature selection pipeline. Each model’s feature importance and input–output relationships were explored using TreeExplainer from the Shapely Additive Explanations explainable machine learning package. The mean $R_{adj}^{2}$ (SD) from fivefold cross-validation on training data and the $R_{adj}^{2}\;$ score on test data were 0.38 (0.04) and 0.41 for UGS, 0.45 (0.04) and 0.46 for MGS, and 0.19 (0.02) and 0.21 for GSR. Each model selected features across all categories. Features common to all models were age, grip strength, chair stands time, mean motor reaction time, and height. Exclusive to UGS and MGS were educational attainment, fear of falling, Montreal cognitive assessment errors, and orthostatic intolerance. Exclusive to MGS and GSR were body mass index (BMI), and number of medications. No features were selected exclusively for UGS and GSR. Features unique to UGS were resting-state pulse interval, Center for Epidemiologic Studies Depression Scale (CESD) depression, sit-to-stand difference in diastolic blood pressure, and left visual acuity. Unique to MGS were standard deviation in sustained attention to response task times, resting-state heart rate, smoking status, total heartbeat power during paced breathing, and visual acuity. Unique to GSR were accuracy proportion in a sound-induced flash illusion test, Mini-mental State Examination errors, and number of cardiovascular conditions. No interactions were present in the GSR model. The four features that overall gave the most impactful interactions in the UGS and MGS models were age, chair stands time, grip strength, and BMI. These findings may help provide new insights into the multisystem predictors of gait speed and gait speed reserve in older adults and support a network physiology approach to their study.

## Introduction

Gait speed is a measure of general fitness (Wu and Zhao, 2021); faster gait speed is associated with the ability to meet occupational demands in younger adults (Aldridge et al., 2020), whilst slower gait speed is associated with functional decline and morbidity in older adults (Bohannon, 1992; Kawajiri et al., 2019). Even though usual (or comfortable) gait speed (UGS) and maximum gait speed (MGS) are significantly intercorrelated (Kollen et al., 2006), changing from comfortable to maximum speed requires a general effort across many body systems. The difference between these two gait speeds has been referred to as walking speed reserve or gait speed reserve (GSR) (Noguerón García et al., 2020).

UGS is a commonly measured gait characteristic in clinical practice and has well-established associations with age (Samson et al., 2001), physical function (James et al., 2020), and frailty (O’Donoghue et al., 2021). On the other hand, MGS has been associated with physical and cognitive function (Umegaki et al., 2018; Aldridge et al., 2020). Gait speed reserve (GSR) may be a useful proxy measure of physiological reserve in humans. For example, some studies have suggested that in community-dwelling older adults, the simultaneous consideration of both usual and maximum gait speed could increase the specificity of the identification of frailty (Noguerón García et al., 2020; do Carmo Correia de Lima et al., 2019). The health associations of these three modalities of gait speed (UGS, MGS, and GSR) are somewhat different, but to our knowledge, there have been no systematic attempts to model predictors of GSR in a large representative sample of community-dwelling older adults where many demographic, anthropometric, and clinical features are measured across multiple physiological systems.

In this study, we aimed to use machine learning to, first, identify the set of features that, from a shortlist of features, best describe UGS, MGS, and GSR. The shortlist of features was theory-driven and not purely exploratory; that is, we selected features that might have physiological plausibility. Then, using explainable machine learning methods, we investigated the selected models for UGS, MGS, and GSR to observe how each feature in the model was associated with the output in a non-parametric manner. With the selected features and visualizations of the input–output relationships, we then discussed the clinical interpretations with respect to the cohort used and the hypothesis that UGS, MGS, and GSR are multisystem phenomena. To touch on the relevance of the gait speed variables with respect to clinical associations, a brief exploration of differences in gait speeds between fallers/non-fallers and fainters/non-fainters was included.

The paper is structured as follows: first, we describe the TILDA study and the methods used to collect data on the shortlisted features; next, the methods used to compare fallers/non-fallers and fainter/non-fainters are described; then, we describe the feature selection pipeline and provide an overview of the histogram gradient boosting regression machine learning model employed. The *Materials and Methods* section ends with a brief description of the Shapley additive explanations (SHAP) package used to explain the models. The results from feature selection, and SHAP interpretation are then presented separately for each of the three models: UGS, MGS, and GSR. Finally, the discussion and conclusions compare the results of each model, and comment on the potential clinical relevance.

## Materials and Methods

### Design and Setting

We analysed data from adults aged 50+ from Wave 3 of TILDA, a population-based longitudinal study of ageing (https://tilda.tcd.ie/). TILDA study design, and the full cohort profile, have been previously described in detail (Kearney et al., 2011a; Donoghue et al., 2018). Wave 3 data collection took place in 2014 and 2015 and included a computer-assisted personal interview conducted by social interviewers in the participants’ home, a self-completion questionnaire completed in the participants’ own time, and a detailed suite of technology-aided health assessments conducted by trained research nurses at a dedicated health assessment centre. Ethical approval was obtained from the Faculty of Health Sciences Research Ethics Committee at Trinity College Dublin, Ireland (Reference: Main Wave 3 Tilda Study; approval date: June 9, 2014). All participants provided written informed consent, and all data collection procedures adhered to the World Medical Association Declaration of Helsinki on ethical principles for medical research involving human subjects.

#### Analytical Sample

The primary analytical sample consisted of participants from TILDA Wave 3 aged 50 years or more who had data for both UGS and MGS.

#### Gait Speed Measures

At Wave 3 of TILDA, gait speed was measured as part of a health centre assessment. Measurements in units of cm/s were made using a 4.88-m computerized walkway (GAITRite, CIR Systems, NY, United States). A 2-m space before and after the walkway was used for acceleration and deceleration. Participants were first asked to walk at their normal (usual) pace, UGS, and then as fast as they safely could, MGS. Two walking trials were obtained in each condition, and the mean value for each was used in this analysis. GSR was defined as MGS–UGS.

#### Falls and Faints

To put UGS, MGS, and GSR into clinical context, we assessed their correlation with both historical and future falls and syncope.

Historical fallers/fainters were defined as participants who reported at least one fall/faint in the year prior to Wave 3.

Future fallers/fainters were defined as those who reported at least one fall/faint between Wave 3 and Wave 5 (approximately 4 years later).

Each of those four variables were binary categorical with occurrence of falls/faints coded as “1” and absence coded as “0.”

#### Shortlisted Features

A shortlist of features from the TILDA Wave 3 dataset was manually curated by the lead author (JD, trained in a STEM discipline), in consultation with co-authors representing both STEM (BH) and health/medical (SK, OD, and RRO) fields. The features were chosen based on known or plausible associations with the three gait speed modalities under investigation. The feature curation considered features from the following five categories: socio-demographics/anthropometrics/medical history, cardiovascular system, physical strength, senses, and cognitive/psychological.

##### Socio-Demographics/Anthropometrics/Medical History

Demographic information included Age in years, Sex (male = 0; female = 1), and level of educational attainment (Edu3): either primary/none (Edu3 = 1), secondary (Edu3 = 2), or tertiary/higher (Edu3 = 3).

Anthropometrics comprised Weight (kg), Height (cm), body mass index (BMI, kg/m^2^), and waist-to-hip ratio (WaistHipRatio, waist circumference/hip circumference) (Nolan et al., 2016).

Medical history: number of cardiovascular diseases (nCVD, from the following list: hypertension, angina, heart attack, congestive heart failure, diabetes, stroke, transient ischaemic attack, high cholesterol, heart murmur, abnormal heart rhythm, atrial fibrillation), if taking any Antidepressant medications (binary), if taking any Antihypertensive medications (binary), and the total number of medications being taken excluding supplements (nMeds) (TILDA, 2016).

Smoking status (Smoker) was categorized as: never (smoker = 0), past (smoker = 1), and current (smoker = 2). Alcohol intake was scored with the CAGE scale (Bush et al., 1987).

The number of reported difficulties with activities of daily living were also assessed. The six basic activities (ADLs) were dressing, including putting on shoes and socks; walking across a room; bathing or showering; eating, such as cutting up food; getting in or out of bed; and using the toilet, including getting up or down. The six independent activities (IADLs) were preparing a hot meal, doing household chores (laundry, cleaning), shopping for groceries, making telephone calls, taking medications, and managing money such as paying bills and keeping track of expenses (Romero-Ortuno et al., 2019).

##### Cardiovascular System

During the TILDA Wave 3 health assessment, resting-state (RS) cardiovascular measurements were made during an approximately 10-min window in which the participant was lying supine in a comfortably lit room at an ambient temperature of between 21°C and 23°C. The full TILDA active stand protocol in which the resting-state window takes place has been detailed elsewhere (Finucane et al., 2014; Knight et al., 2020; Donoghue et al., 2021). Throughout the RS, participants underwent non-invasive continuous haemodynamic monitoring using a Finometer MIDI device (Finapres Medical Systems BV, Amsterdam, Netherlands). All RS parameters selected for the shortlist are mean values from the last minute of supine rest (Knight et al., 2020). Haemodynamic parameters were systolic blood pressure (sBP_RS), diastolic blood pressure (dBP_RS), mean arterial pressure (MAP_RS) all in units of mmHg , heart rate (HR_RS) in bpm , stroke volume (StrokeVolume_RS) in ml , left ventricular ejection time (LVET_RS) in ms , pulse interval (PulseInterval_RS) in ms , maximum slope (Maxslope_RS) in mmHg/s , cardiac output (CardiacOutput_RS) in L/min , and total peripheral resistance (TPR_RS) in $\text{dyn}\cdot \text{s}\cdot {\text{cm}}^{-5}$ . A near-infrared spectroscopy (NIRS) device, attached over the participants’ left frontal lobe area, was also employed during the RS, and the following cerebral oxygenation features were extracted, again as the mean values from the final minute of rest: oxygenated haemoglobin concentration (O2Hb_RS) and deoxygenated haemoglobin concentration (HHb_RS) both in units of μmol/L , and tissue saturation index (TSI_RS) as a percentage (Knight et al., 2020). Previously derived sample entropy values for resting sBP (sBP_RS_SampEn), dBP (dBP_RS_SampEn), MAP (MAP_RS_SampEn), heart rate (HR_RS_SampEn), O2Hb (O2Hb_RS_SampEn), HHb (HHb_RS_SampEn), and tissue saturation index (TSI_RS_SampEn) were also shortlisted (Knight et al., 2020). In addition, participants were asked if they experienced dizziness upon standing (PhasicDizziness, yes or no), and this feature was also included in the shortlist.

Resting heart rate variability measures were also shortlisted; these were obtained in two 5-min blocks as detailed elsewhere (Frewen et al., 2013). In short, for each block, participants were lying supine. In the first block, participants were asked to breath spontaneously (free breathing), and in the second block, they were asked to breath according to a pre-recorded set of auditory instructions (paced breathing at a frequency of 0.2 Hz). Measurements were obtained using three-lead electrocardiograms (Medilog Darwin, Oxford Instruments Medical Ltd., United Kingdom). The data were subject to a 0.01–1,000 Hz band-pass filtering before R peak detection was performed with a proprietary software (Pardey and Jouravleva, 2004). The data collection and processing are described in detail elsewhere (Frewen et al., 2013). Time domain features were mean heart rate in bpm, root mean square of successive differences between RR intervals in ms, standard deviation of NN intervals in ms, and difference between maximum and minimum heart rate in bpm, derived for both free (HR_Mean_Free, HR_rMSSD_Free, HR_SDNN_Free, HR_Span_Free) and paced breathing (HR_Mean_Paced, HR_rMSSD_Paced, HR_SDNN_Paced, HR_Span_Paced). The difference between free and paced breathing values was calculated for rMSSD (HR_rMSSD_PacedFreeDiff). In the frequency domain, total spectral power in the 0–0.4 Hz frequency band was measured for both free (HR_TotalPower_Free) and paced breathing (HR_TotalPower_Paced) in units of milliseconds squared (ms^2^).

sBP, dBP, and HR were also determined in a more conventional manner using a sphygmomanometer in seated (sBP_Seated, dBP_Seated, and HR_Seated) and standing (sBP_Standing, dBP_Standing, and HR_Standing) positions, all with units of mmHg. The difference between seated and standing values were calculated for each of the measures (sBP_SeatStandDiff, dBP_SeatStandDiff, and HR_SeatStandDiff).

Pulse wave velocity (PulseWaveVelocity), a non-invasive measure of arterial stiffness with units of m/s, was also included as a cardiovascular feature. In TILDA, the average of two measurements between the carotid and femoral arteries (in m/s) was obtained using a Vicorder^®^ (SMT medical GmbH & Co., Wuerzburg, Germany). Full details have been described elsewhere (Nolan et al., 2016; Donoghue et al., 2018).

##### Physical Strength

Upper and lower body strengths were assessed *via* grip strength and chair stands time. Grip strength was measured in kg using a hydraulic hand dynamometer (Baseline^®^, Fabrication Enterprises, Inc., White Plains, NY, United States). The value for grip strength referred to henceforth (GripStrength) is the maximum value from a total of eight measurements with four made on each hand. Lower body strength was assessed using the chair stands test in which the time (in seconds) was recorded for the participants to complete five chair stands as quickly as possible, keeping the arms folded across their chest (ChairStandsTime). Chair height was 46 cm.

##### Cognitive and Psychological

Global cognition was assessed using two paper-based assessments: the Montreal Cognitive Assessment (MOCA) (Nasreddine et al., 2005) and the Mini-Mental State Examination (MMSE) (Arevalo-Rodriguez et al., 2015); from these, the number of errors (MOCA_errors and MMSE_errors) were extracted for the feature shortlist. Concentration, cognitive processing, and motor response were assessed using two computer-assisted tasks: the choice reaction task (Chintapalli and Romero-Ortuno, 2021) and the sustained attention to response task (SART) (O'Halloran et al., 2014). The choice reaction task required participants to hold down a central button until an on-screen stimulus (either the word “YES” or “NO”) appeared, at which time they had to press the corresponding button on a keyboard. After pressing either button, participants were then required to return to the central button to continue. This was repeated approximately 100 times. In the SART test, participants watched a screen that displayed the numbers 1–9 sequentially a total of 23 times. A number appeared for 300 ms with an interval of 800 ms between numbers: the entire trial lasts approximately 4 min. Participants were instructed to press a button at the appearance of every number except for a specific number (i.e., 3). We extracted the following features from the choice reaction task: mean and standard deviation of cognitive reaction time (CRT_mean and CRT_SD) and motor response time (MRT_mean and MRT_SD) and the number of correct CRT presses (CRT_correct). CRT is the time taken to release the central button in response to the stimulus; MRT is the time between releasing the central button and pressing the required button. From the SART, we extracted the following: mean and standard deviation of reaction time (SART_mean and SART_SD) and the number of trials in which the participant pressed the button when the number 3 appeared (SART_errors). CRT, MRT, and SART times are all measured in milliseconds.

The psychological domains of depression, anxiety, and loneliness were assessed using the Center for Epidemiologic Studies Depression Scale (CESD), the Hospital Anxiety and Depression Scale—Anxiety subscale (HADSA), and the UCLA Loneliness Scale (UCLA), respectively. Fear of falling (FOF) was determined with a yes or no question (Donoghue et al., 2018).

##### Sensory

Visual acuity (VA) was measured using a LogMar chart. VA in the left eye (VisualAcuityLeft), right eye (VisualAcuityRight), and best VA (VisualAcuityBest) were included in this work. VA left and right were in logarithmic units. Best VA was defined as $100-\left(\text{min}(\left[VA_{left},\;\;VA_{right}\right]\right)\times 50$ . Contrast sensitivity (CS) was measured at five spatial frequencies; in cycles per degree (cpd), they were 1.5 cpd (cs_score_a), 3 cpd (cs_score_b), 6 cpd (cs_score_c), 12 cpd (cs_score_d), and 18 cpd (cs_score_e). The procedures for visual acuity and contrast sensitivity measurements are described in detail elsewhere (Duggan et al., 2017). Self-reported hearing (Hearing_SR) was ascertained by the question: “Is your hearing (with or without a hearing aid): 1. Excellent, 2. Very good, 3. Good, 4. Fair, or, 5. Poor?”

Multisensory integration was measured using the Shams sound-induced flash illusion (SIFI) test (Shams et al., 2002). The procedure used in TILDA is described in more detail elsewhere (Hirst et al., 2021), but in short, participants were subjected to a set of beeps and flashes and asked to report how many flashes they perceived. Five general flash–beep combinations were presented to the participants: two beeps + two flashes; one beep + one flash; zero beeps + one flash; zero beeps + two flashes; and two beeps + one flash. The flash–beep configurations used in this analysis are the so-called “illusory” two-beep one-flash (2B1F) trials. In 2B1F trials, the flash is synchronous with one of the beeps; the other beep occurred either 70, 150, or 230 ms before (SIFI_2B1F_70, SIFI_2B1F_150, SIFI_2B1F_230) or after (SIFI_2B1F_m70, SIFI_2B1F_m150, and SIFI_2B1F_m230) the flash–beep pair. SIFI susceptibility represented accuracy for judging how many flashes were presented when one flash was presented with two beeps (2B1F). Lower accuracy, judging one flash as two, thus indicates higher SIFI susceptibility and stronger integration. SIFI susceptibility was expressed as proportion correct. As there were two trials per condition, these variables were considered discrete (i.e., participants scored 0, 0.5, or 1 proportion correct) (Hirst et al., 2020).

### Statistical Association Between Gait Speed Modalities and Faller/Fainter Status

The normality of the distribution of the three gait speed variables was determined using the one-sample Kolmogorov–Smirnoff test. All three gait speed variables resulted to be non-normally distributed. Hence, to examine the bivariate associations between UGS, MGS, and GSR and historical and future occurrence of falls and faints, we utilized the non-parametric two-sided independent samples Mann–Whitney *U*-test.

### Overview of Machine Learning Steps

A general overview of the machine earning steps are as follows. The machine learning regression model employed is called histogram gradient boosting regression. In a stepwise fashion, features are tried out one by one in this model, and the best one is selected; this step is repeated over and over until the model does not get any better. The final model is trained on the set of features that give the best performance. This final model is then passed through an explainable machine learning step whereby a method from the SHAP package called TreeExplainer is used to observe the relationships between each of the features in the model and the output of the model.

### Histogram Gradient Boosting Regression

The regression model employed for this analysis was the histogram gradient boosting regressor (HGBR) from Scikit-learn (Pedregosa et al., 2012) version 0.24. The Scikit-learn implementation is based on Microsoft’s light gradient boosting machines (Ke et al., 2017).

Gradient boosting (Friedman, 2001) is a machine learning technique that builds decision trees sequentially where each one is constructed such that it predicts the residuals from the previous tree. Gradient boosting is a powerful tool that has become the model of choice in many fields and applications (Chen and Guestrin, 2016) and have been shown to outperform deep-learning models where the data are tabular and the features themselves have individual meanings as opposed to data structured in a temporal and/or spatial manner as is the case for problems in image and audio domains (Lundberg et al., 2020). Light gradient boosting machines and histogram gradient boosting is an adaptation of gradient-boosted trees that places feature values into histogram-like bins, which allow for tree split points to be located more efficiently.

The HGBR model inherently supports missing values and categorical data. The support for missing data helps to avoid the need for data imputation or removal of features. The categorical data support avoids the need for dummy variables and one-hot encoding, which can drastically increase the dimensionality of the input feature space.

### Feature Selection Algorithm

All operations were performed using Python 3. The feature selection was executed on the Tinney High Performance Computing Cluster at Trinity College Dublin (https://www.tchpc.tcd.ie/node/1353).

Prior to any feature selection or training of any kind, the data were divided according to an 80/20 train/test split. From the shortlisted set of 88 features across 5 domains, features were chosen for the final models using an automated stepwise feature selection algorithm. In this algorithm, each feature is individually added to a temporary model that contains all features previously selected for the final model: for the initial round, each temporary model contains a single feature. For each individual temporary model, a hyperparameter tuning is performed in which a fivefold cross-validation (CV) is used on the training data for each set of hyperparameters. The hyperparameter tuning is in the form of a 100-iteration randomized search of a set of predefined hyperparameter distributions:

{‘max_iter’: [2000],
‘loss’: [‘least_squares’],
‘random_state’: [42],
‘early_stopping’: [True],
‘learning_rate’: loguniform(0.005, 0.1),
‘max_leaf_nodes’: randint(2, 10),
‘min_samples_leaf’: randint(100,200)}

The evaluation metric employed was adjusted $R^{2}$ ( $R_{adj}^{2}$ ). This metric is used to avoid the continual increase in $R^{2}$ that occurs with the addition of new features regardless of whether they significantly increase the variance explained by the model. For each temporary model, the best parameters are chosen based on the mean $R_{adj}^{2}$ from CV ( $\bar{R_{adj}^{2}})$ . From these temporary models, the one that provides the biggest increase in $\bar{R_{adj}^{2}}$ is chosen to continue with, i.e., the new feature upon which that temporary model is based is added to the final model. Before moving to the selection of the next feature, each feature in the model is removed one by one to check if any of them have become redundant in light of the addition of the newest feature; if the score improves on removing a feature, then that feature is removed from the model.

For the purpose of performance monitoring, on each iteration of the loop, the current best model is fit to the entire training dataset and evaluated on both the training and test sets to give training and test $R_{adj}^{2}$ scores. These scores do not influence the feature selection.

### SHapley Additive exPlanations Values

In this work, SHapley Additive exPlanations (SHAP) values (Lundberg and Lee, 2017) were employed to assess feature importance and investigate the impact of features on the model output. Specifically, the TreeExplainer method from the SHAP package is used. TreeExplainer is designed for use with tree-based machine learning models and builds interpretations that are theoretically guaranteed to be faithful at the local and global levels (i.e., the level of individual samples and the level of features as a whole) (Lundberg et al., 2020).

Shapley values, from which the SHAP package derives, were presented in the field of cooperative game theory (Shapley et al., 1953). They guarantee a fair distribution of contributions from each feature in a model. However, it is generally NP hard (i.e., complexity and computation time scales exponentially with number of features) to compute them, and as such, they have not been widely utilized. The SHAP package first developed a model agnostic heuristic that allowed for their use. A more recent development allows for exact Shapley values to be computed for tree-based models in a practical, low-order polynomial time. TreeExplainer is designed such that it does not need to compute Shapley values for the entire feature set but instead uses the tree structure to perform the exact computation on smaller feature sets made possible by the tree. Detailed derivations of Shapley values and of the TreeExplainer algorithm (Lundberg et al., 2020) can be found elsewhere, but briefly, Shapley derived these values as a method of attributing worth to each player in a game in a fair way. In coalitional game theory, n players form a grand coalition, S, that has a total worth, ${\text{Δ}}_{S}$ . Each player is representative of an input feature. Each smaller coalition, Q ; Q⊂S , has worth ${\text{Δ}}_{Q}$ . A Shapley value is a unique solution that satisfies the following four axioms developed to ensure a fair distribution of worth:

1. The sum of contributions from each player equals the total worth of the game.
2. If a coalition W not containing player i has a worth equal to that of coalition W in union with player i , then the worth of player i is zero; i.e., the player i did not increase the worth of coalition W .
3. If the worth of coalition W in union with player i is equal to the worth of W in union with j , the worth of player j is equal to the worth of player j .
4. For players w and x , the contribution of a single feature for the sum of values from players w and x is equal to the sum of the contributions from that feature having values of w and x , where the same is true for any subset *Q* of features and instances *w* and *x.* Said in another way, for a feature, *f*, and values for *f* of *x* and *w*, the contribution ${\text{Δ}}_{Q}\left(f=x+w\right)$ is equal to the contribution of ${\text{Δ}}_{Q}\left(\left(f=x\right)+\left(f=w\right)\right)$ .

SHAP values are computed for each sample of each feature. This allows for global feature explanations to be constructed from the sample level either visually in the form of SHAP summary plots or as a single value such as mean absolute SHAP value or maximum absolute SHAP value. The nature of SHAP values being true to local impacts of features means that low-frequency, high-impact effects do not go unnoticed. For example, a particular feature might, for most samples, have a low impact; however, for some small subset of samples, the feature might have a very large impact. SHAP interaction values are also readily available that explain the impact of interactions between two features. SHAP values are presented as having a positive or negative impact on the output of the model with respect to the expected model output, i.e., the mean output of the model. Thus, for an individual sample, the SHAP value for a particular feature might be, for example, −2.5; this should be interpreted as the value of that feature for that sample is associated with a model output that is −2.5 units less than the model’s mean output.

All SHAP values shown in the results are for the test data.

## Results

Note on presentation of results: the method for feature selection describes a situation whereby features can be removed from the model if they are made redundant by the addition of new features; this did not occur in any of the models, and as such, all features named henceforth with regard to feature selection are to be understood as features added to the model.

### Analytical Cohort

**FIGURE 1:**
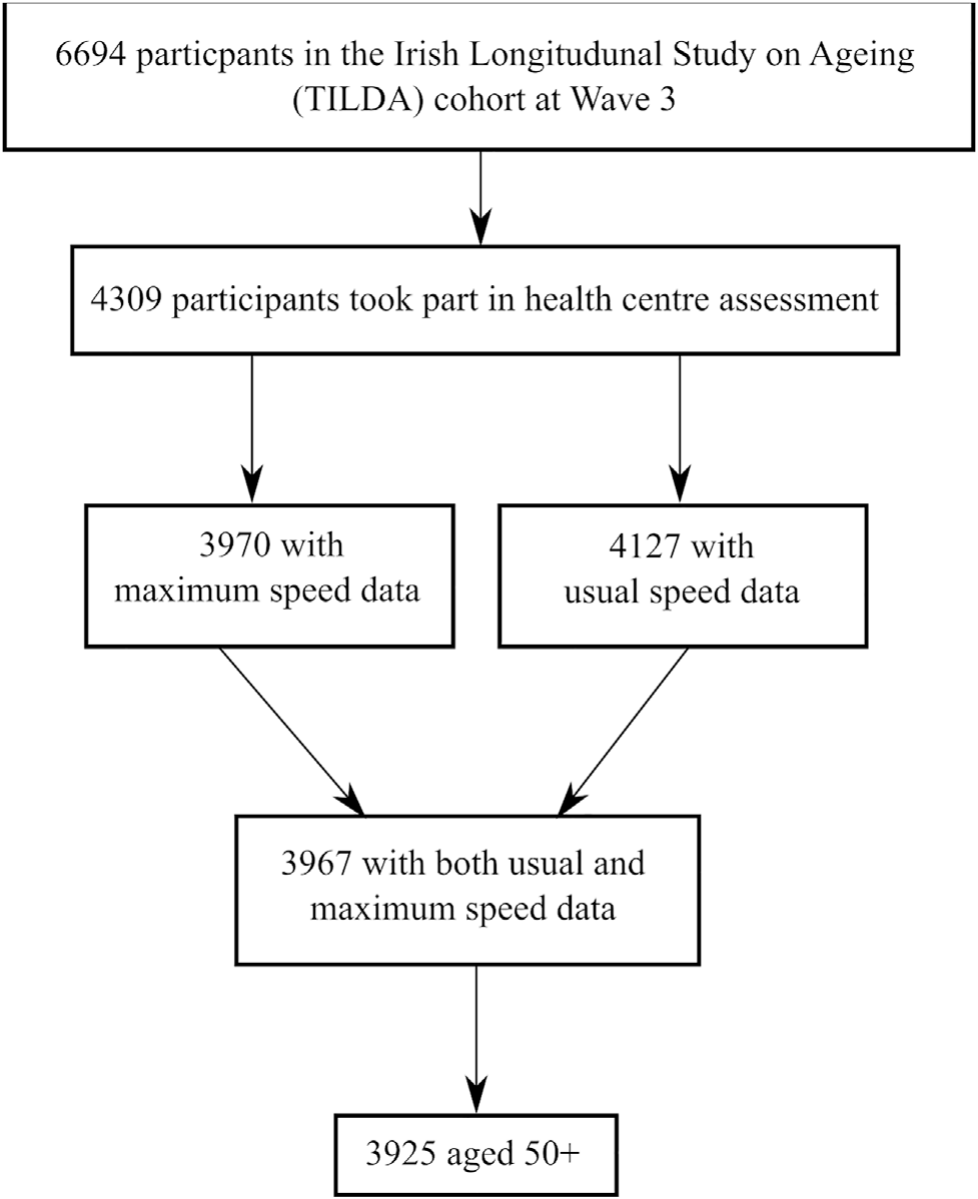
Analytical sample inclusion flowchart.

In TILDA Wave 3, 4,309 participants completed the health centre assessment (Donoghue et al., 2018), where the gait speed tests were conducted. After exclusion of participants aged <50 years or with missing data for either UGS or MGS, there were *N* = 3,925 participants, with 2,156 (55%) being female. An analytical sample inclusion flowchart can be seen in Figure 1. The educational attainment breakdown was as follows: third/higher, 1,685 (43%); secondary, 1,571 (40%); and primary/none, 669 (17%). The analytical cohort had a mean (SD) age of 64.5 (7.8) years, UGS of 136.7 (19.2) cm/s, MGS of 171.0 (26.9) cm/s, and GSR of 34.3 (16.6) cm/s.

### Group Differences in Faller and Fainter Status

Of the Wave 3 participants, 21.3% were historical fallers, 3.8% historical fainters, 31.9% future fallers, and 5.4% future fainters. Table 1 shows the results of the association between UGS, MGS, and these clinical variables. Differences between historical fallers were all statistically significant, with a largest median difference for MGS. Statistical significance of *p* < 0.05 was demonstrated in historical fainters for UGS and MGS only, with the largest difference also for MGS. A similar pattern emerged for future fallers and fainters.

**TABLE 1:** Group statistics and results of independent samples Mann–Whitney *U*-test for historical and future falls and faints occurrence.

Historical falls and faints								
	Non-fallers [median (IQR)]	Fallers [median (IQR)]	Difference in group median	Mann–Whitney *p*-value	Non-fainters [median (IQR)]	Fainters [median (IQR)]	Difference in group median	Mann–Whitney *p*-value
UGS (cm/s)	139.3 (24)	133.1 (26)	6.2	<0.001	138.3 (25)	133.0 (19)	5.3	0.005
MGS (cm/s)	174.5 (33)	166.2 (36)	8.3	<0.001	172.55 (34)	165.95 (34)	6.6	0.010
GSR (cm/s)	33.0 (20)	30.4 (22)	2.6	<0.001	32.3 (20)	28.95 (23)	3.35	0.118
**Future falls and faints**								
	Non-fallers [median (IQR)]	Fallers [median (IQR)]	Difference in group median	Mann–Whitney *p*-value	Non-fainters [median (IQR)]	Fainters [median (IQR)]	Difference in group median	Mann–Whitney *p*-value
UGS (cm/s)	139.7 (23)	134.8 (27)	4.95	<0.001	138.4 (24)	134.1 (30)	3.7	<0.001
MGS (cm/s)	175.1 (33)	168.0 (36)	7.05	<0.001	173.2 (33)	165.7 (39)	6.0	<0.001
GSR (cm/s)	33.20 (21)	31.4 (21)	1.85	<0.001	32.7 (21)	31.1 (21)	1.6	0.209

### Usual Gait Speed

**FIGURE 2:**
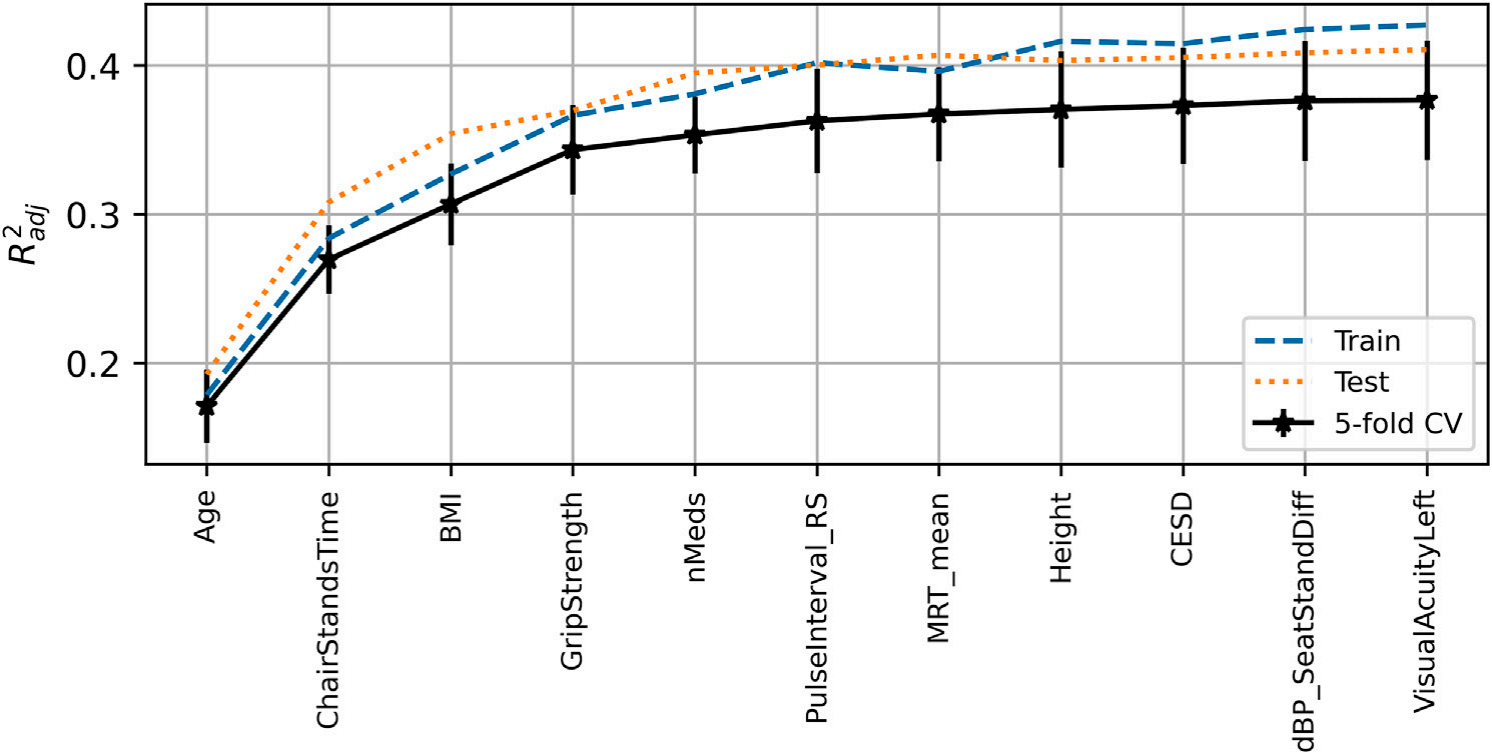
Visualization of the feature selection process for the usual gait speed model. From left to right on the *x*-axis, the features are in order of addition to the model. The *y*-axis shows the dimensionless $R_{\mathrm{adj}}^{2}$ metric. Mean fivefold cross-validation scores with error bars showing ± SD are shown in black, train scores in dashed blue, and test scores in dotted red.

The peak $\bar{R_{adj}^{2}}\left(SD\right)$ achieved for the UGS model was 0.38 (0.04) with training and test scores of 0.43 and 0.41, respectively. The expected model output was 136.6 cm/s. The features chosen for the model, in order of selection as per Figure 2, were age, chair stands time, BMI, grip strength, number of medications, resting-state pulse interval, mean motor reaction time, height, depression score, sit-to-stand difference in diastolic blood pressure, and left visual acuity.

**FIGURE 3:**
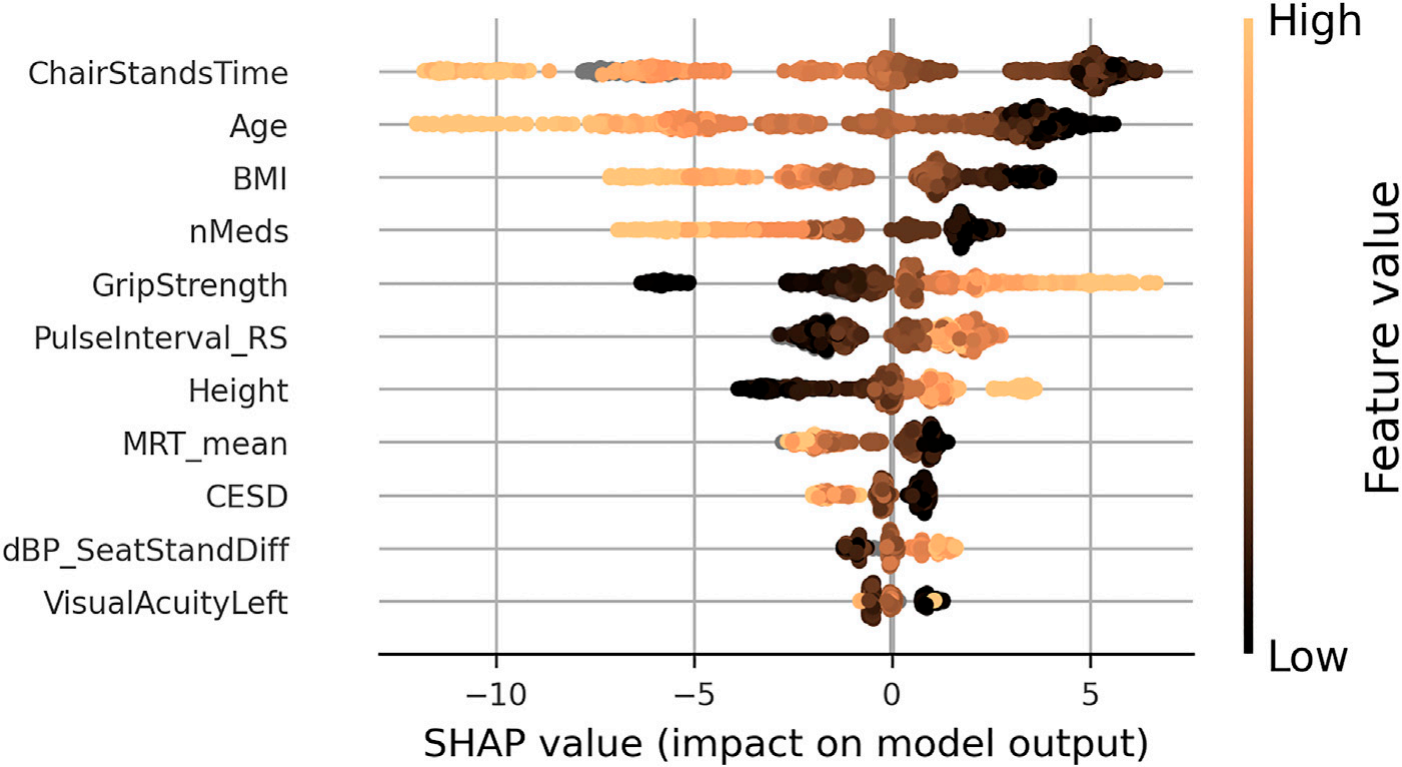
SHAP summary plot for the final usual gait speed model. Features are ordered from top to bottom by decreasing mean absolute SHAP value. For each feature, each point represents a single sample in the test data. A sample’s x-coordinate displays the SHAP value for that sample with respect to a given feature. The colour of a sample indicates the value of the feature, with light brown being high, black low, and grey missing.

A SHAP summary plot is shown in Figure 3; each point on the x-coordinate represents a samples SHAP value, and its colour signifies the value of the feature for that sample, with light brown being high, black being low, and nan (missing) values appearing grey. On the *y*-axis, features are arranged from top to bottom in order of decreasing mean absolute SHAP value: chair stands time, age, body mass index, number of medications, grip strength, resting-state pulse interval, height, mean motor reaction time, CESD depression score, difference in seated and standing diastolic blood pressure, and visual acuity in the left eye. The figure suggests that upper limits (light brown) of certain variables (e.g., chair stands time, age, body mass index, and number of medications) are more negatively impactful than their lower limits, which are positively impactful. The opposite is the case for upper limits of grip strength, for example.

**FIGURE 4:**
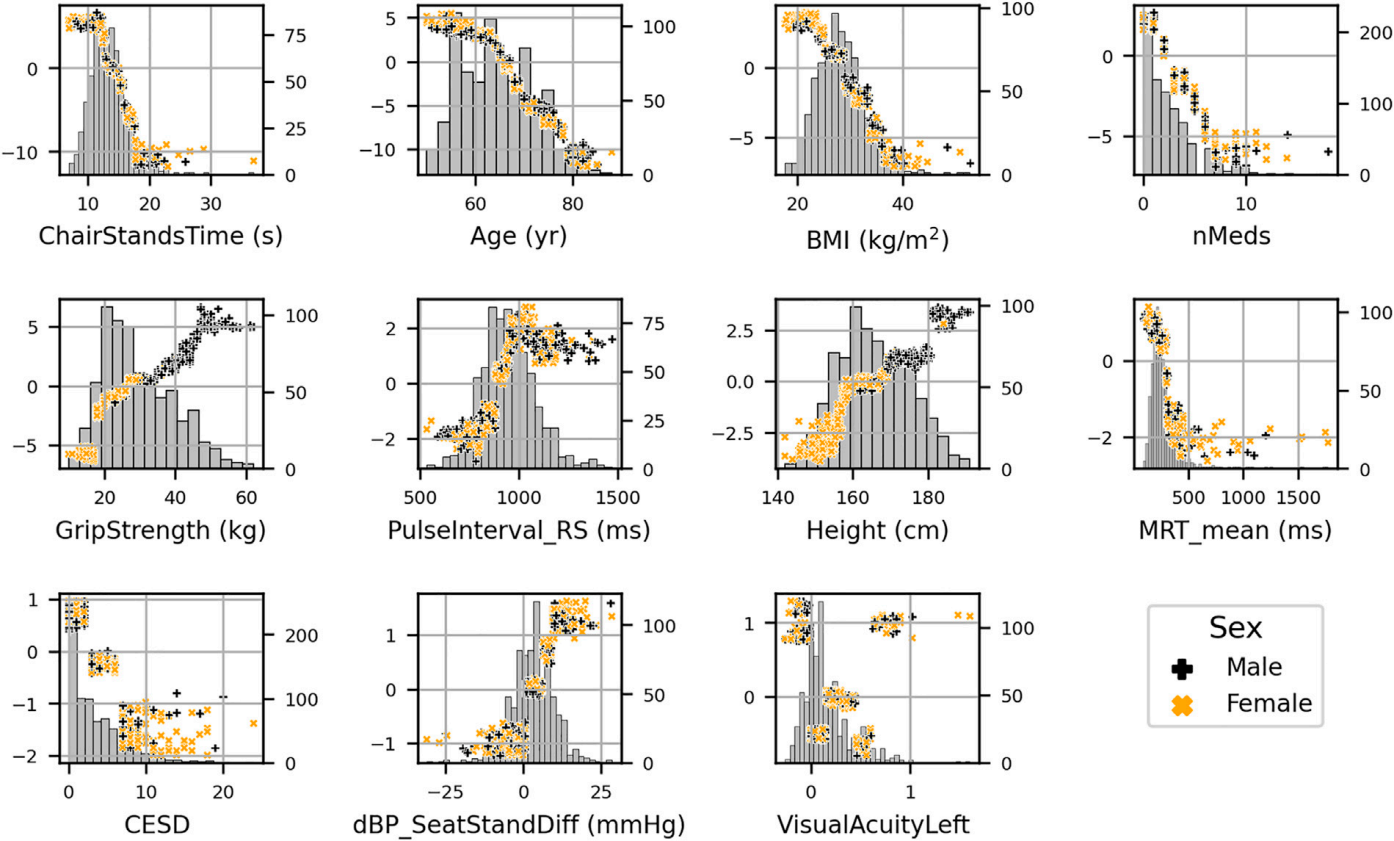
SHAP values (left *y*-axis) vs. input feature value (x- axis) with underlaid histogram (right *y*-axis showing histogram counts) for each feature in the usual gait speed model. Features are arranged top to bottom and left to right in order of decreasing mean absolute SHAP value. Points are coloured per sex: male is black “+,” and female is orange “x.”

Scatter plots of SHAP value vs. feature can be seen for all features in Figure 4. SHAP values (left *y*-axis) vs. input feature value (x- axis) with underlaid histogram (right *y*-axis shows histogram counts) are shown for each feature in the UGS model. Features are arranged top to bottom and left to right in order of decreasing mean absolute SHAP value. At the zero point on the left *y*-axis (SHAP value = 0), the corresponding x-coordinate values for that feature are associated with having no impact on the model (i.e., they are associated with the mean model output). The vertical spread observed in the SHAP values vs. input feature plots indicates the presence of interaction effects. Although not chosen for the model, the data points are coloured by sex.

**FIGURE 5:**
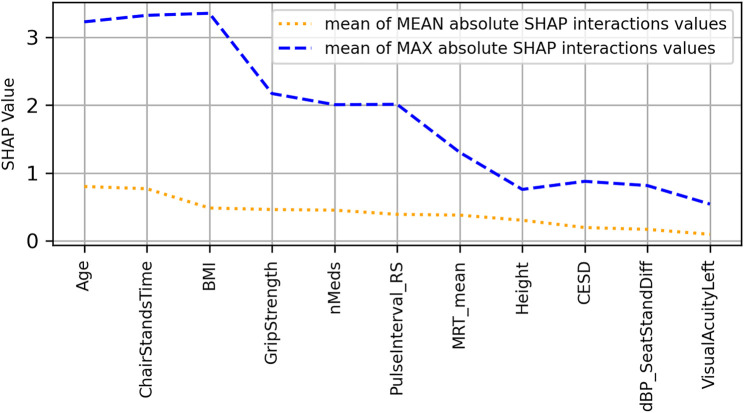
Features ranked from left to right in order of decreasing mean absolute SHAP interaction values (orange dotted line) for the usual gait speed model. Also shown by blue dashed line are the mean maximum absolute SHAP interaction values, which can highlight the effects of outliers.

To further investigate the interaction effects suggested by vertical spreading in Figure 4, a plot (Figure 5) of features ordered by decreasing mean absolute SHAP interaction value was produced; in it, features are ranked from left to right in order of decreasing mean absolute SHAP interaction values (orange dotted line). Also shown in dashed blue are the mean maximum absolute SHAP interaction values, which can highlight the effects of outliers.

The scatter plots of the top 4 interaction effects in the model (i.e., age, chair stands time, body mass index, and grip strength) are shown in Supplementary Appendix A. In the scatter plots, the points are coloured according to the value of the main interaction feature. The interactions are computed for the features in whatever numerical form they exist in, but for ease of visualization, continuous features are coloured according to what quartile a particular samples value falls in; black indicates that the value is in the lowest quartile and light brown the highest quartile. In each figure, the subplots are ordered from top–left to bottom–right by decreasing mean absolute SHAP interaction value.

### Maximum Gait Speed

**FIGURE 6:**
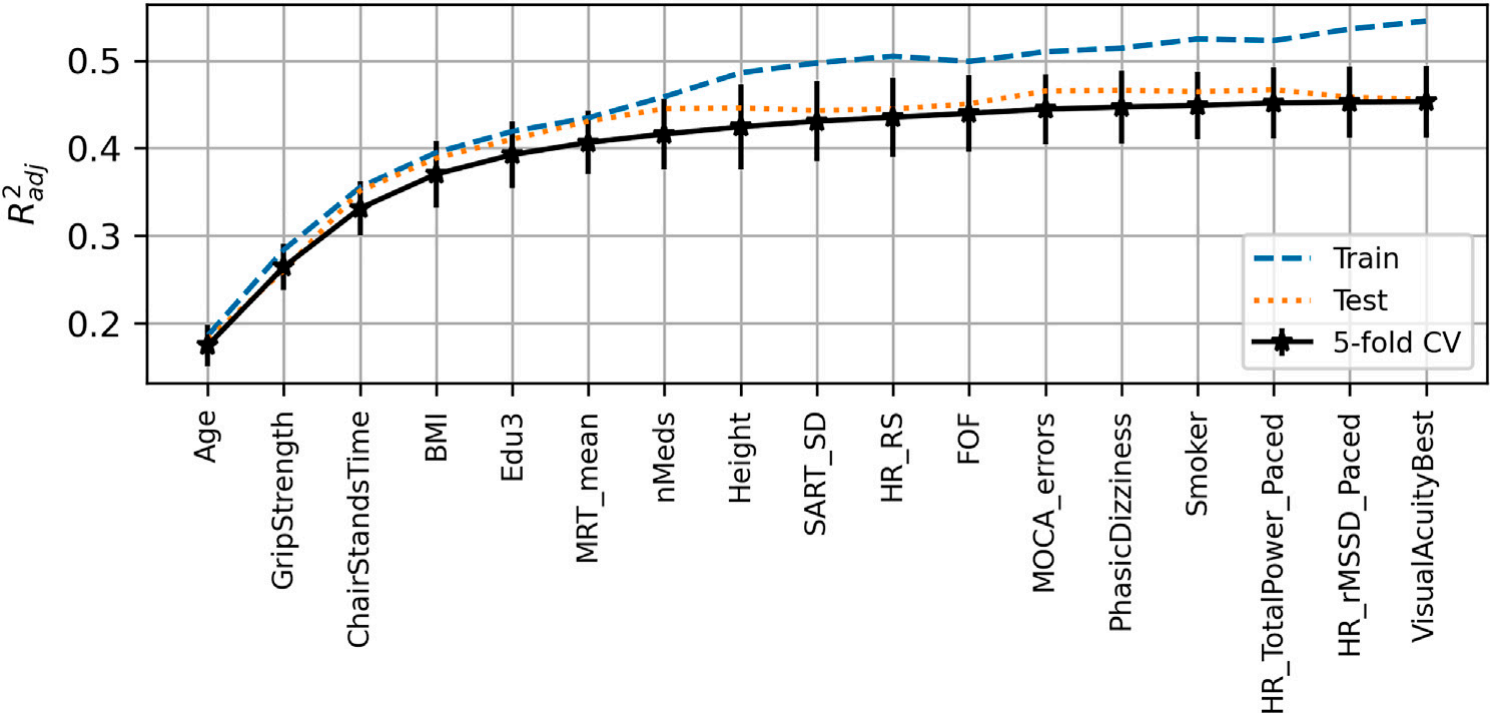
Visualization of the feature selection process for the maximum gait speed model. From left to right on the *x*-axis, the features are in order of addition to the model. The *y*-axis shows the dimensionless $R_{\mathrm{adj}}^{2}$ metric. Mean fivefold cross-validation scores with error bars showing ± SD are shown in black, train scores in dashed blue, and test scores in dotted red.

The peak $\bar{R_{adj}^{2}}\left(SD\right)$ achieved for the MGS model was 0.45 (0.04), with training and test scores of 0.54 and 0.46, respectively. The expected model output was 170.9 cm/s. Features chosen for the model, in order of selection were, age, grip strength, chair stands time, body mass index, education, mean motor reaction time in the choice reaction time test, number of medications, height, the standard deviation of the mean reaction time in the sustained attention to response task, resting-state heart rate, fear of falling, MOCA errors, orthostatic intolerance during active stand, smoking status, total power of the heart rate during paced breathing, the root mean square of successive differences between heartbeats during paced breathing, and best visual acuity. Figure 6 shows the visualization of the feature selection process for this model.

**FIGURE 7:**
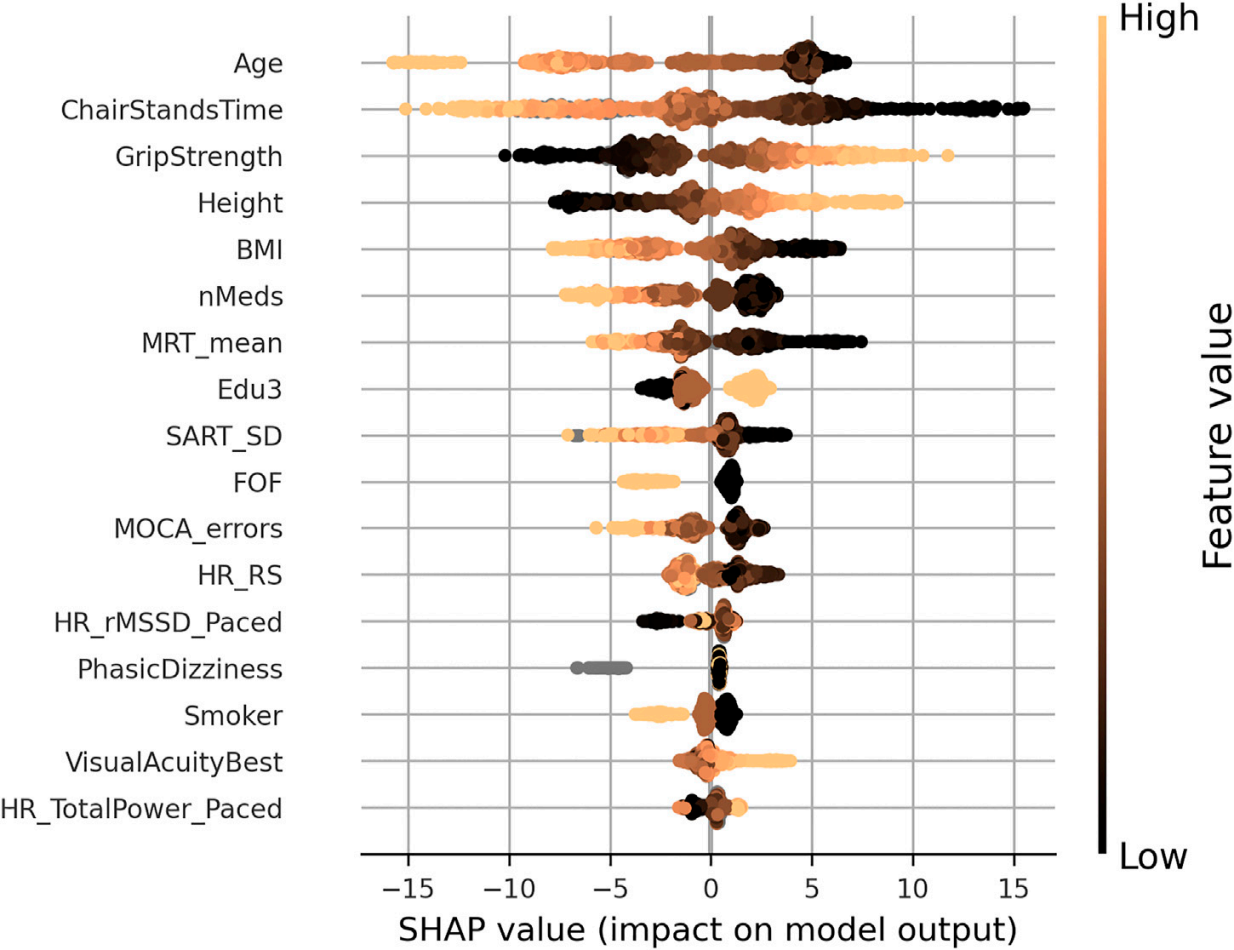
SHAP summary plot for the maximum gait speed model. Features are ordered from top to bottom by decreasing mean absolute SHAP value. For each feature, each point represents a single sample in the test data. A sample’s x-coordinate displays the SHAP value for that sample with respect to the given feature. The colour of a sample indicates the value of the feature, with light brown being high, black low, and grey missing.

In the SHAP summary plot for the MGS model shown in Figure 7, the feature importance ranked in order of decreasing mean absolute SHAP values was age, chair stands time, grip strength, body mass index, height, number of medications, mean motor reaction time in the choice reaction time test, orthostatic intolerance during active stand, education, the standard deviation of the mean reaction time in the sustained attention to response task, fear of falling, MOCA errors, smoking, mean heart rate pre-active stand, the root mean square of successive differences between heartbeats during paced breathing, visual acuity, and total power of the heart rate during paced breathing.

**FIGURE 8:**
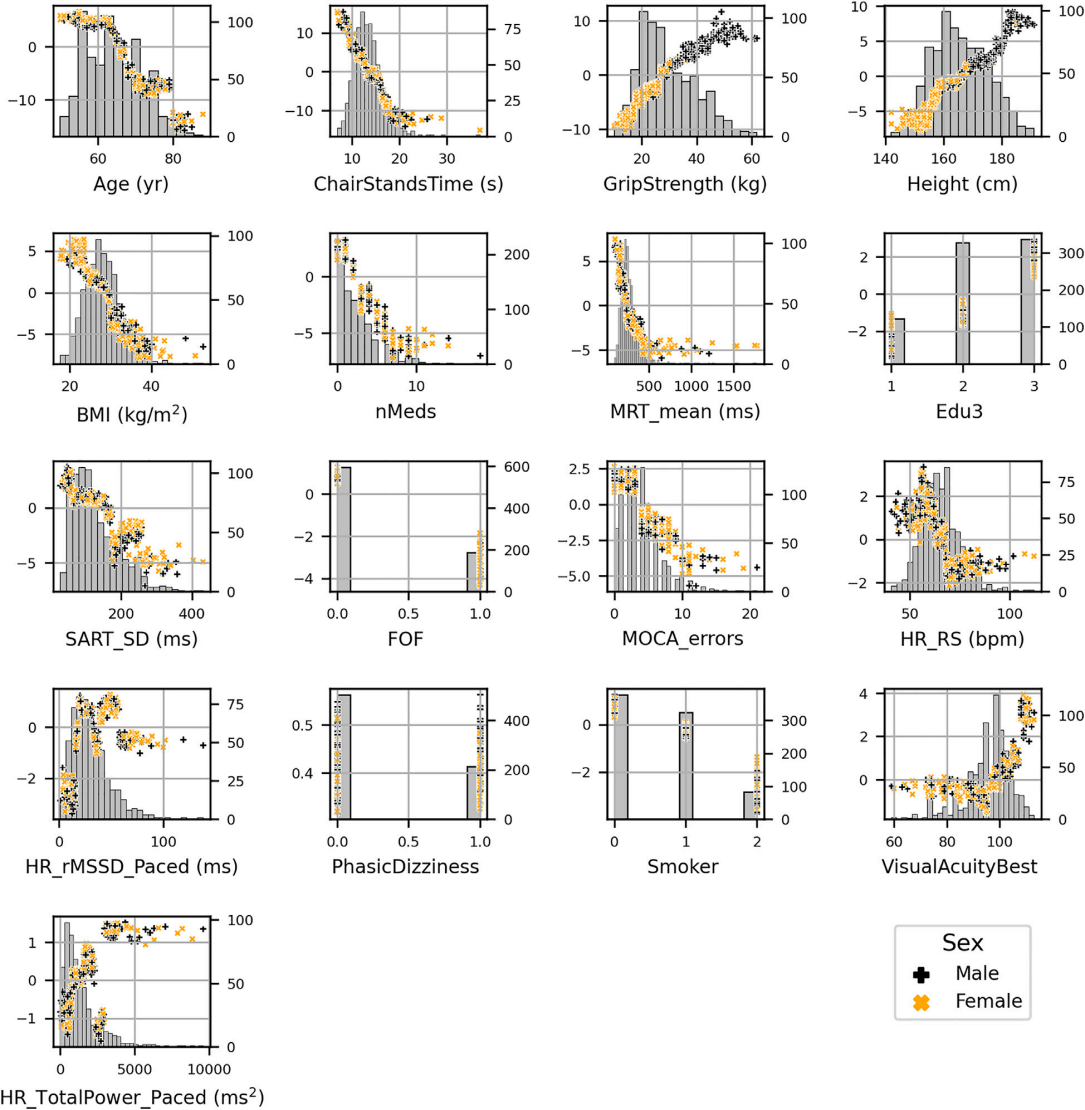
SHAP values (left *y*-axis) vs. input feature value (*x*-axis) with underlaid histogram (right *y*-axis shows histogram counts) for each feature in the maximum gait speed model. Features are arranged top to bottom and left to right in order of decreasing mean absolute SHAP value. Points are coloured per sex: male is black “+,” and female is orange “x.”

**FIGURE 9:**
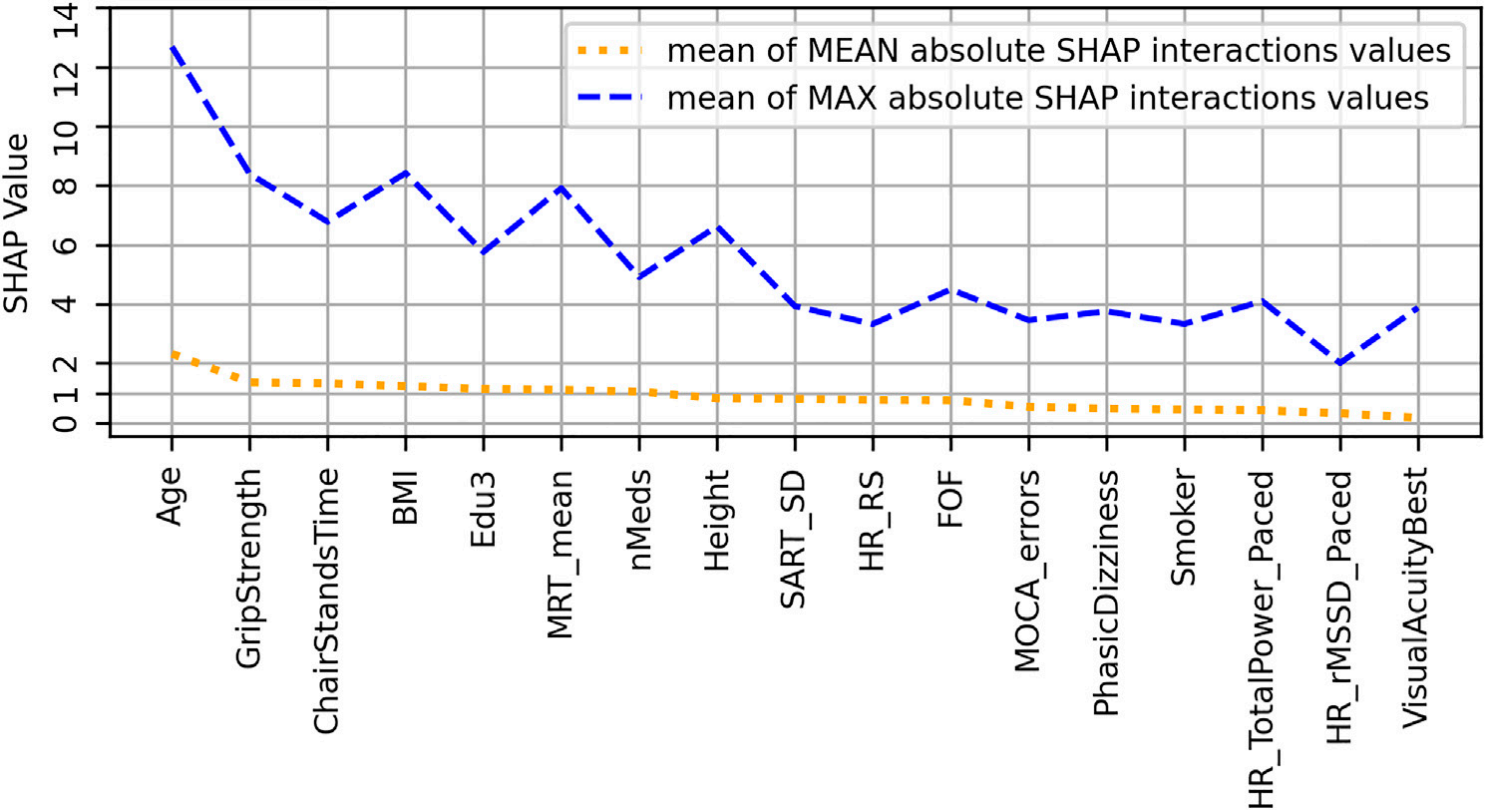
Features ranked from left to right in order of decreasing mean absolute SHAP interaction values (orange dotted line) for the maximum gait speed model. Also shown by blue dashed line are the mean maximum absolute SHAP interaction values, which can highlight the effects of outliers.

Figure 8 shows the SHAP values versus input feature values with underlaid histogram for each feature in the MGS model. Figure 9 shows a plot of features ordered by decreasing mean absolute SHAP interaction value, and Supplementary Appendix B contains the scatter plots of the top four interaction effects in the model.

### Gait Speed Reserve

**FIGURE 10:**
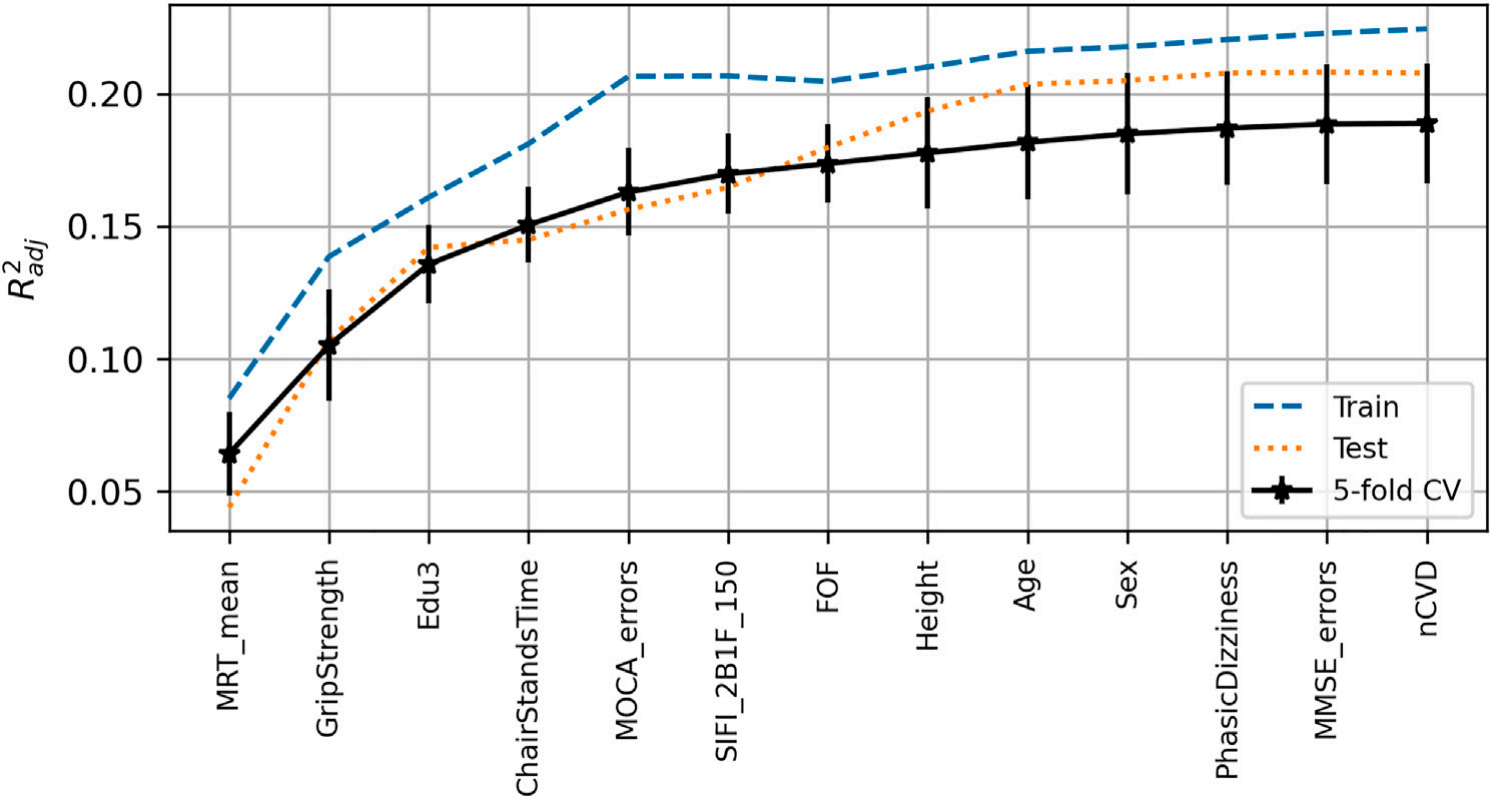
Visualization of feature selection process for gait speed reserve. From left to right on the *x*-axis, the features are in order of addition to the model. The *y*-axis shows the dimensionless $R_{\mathrm{adj}}^{2}$ metric. Mean fivefold cross-validation scores with error bars showing ± SD are shown in black, train scores in dashed blue, and test scores in dotted red.

The peak $\bar{R_{adj}^{2}}\left(SD\right)$ achieved for the GSR model was 0.19 (0.02), with training and test scores of 0.22 and 0.21, respectively. The model expected output was 34.2 cm/s. Figure 10 shows the visualization of the feature selection process. In order of selection, the features chosen were mean motor reaction time in the choice reaction time test, grip strength, education, chair stands time, MOCA errors, accuracy proportion in the sound induced flash illusion (two beeps and one flash with stimulus-onset asynchrony of +150 ms), fear of falling, height, age, sex (0 = male; 1 = female), orthostatic intolerance in the active stand test, MMSE errors, and number of cardiovascular conditions.

**FIGURE 11:**
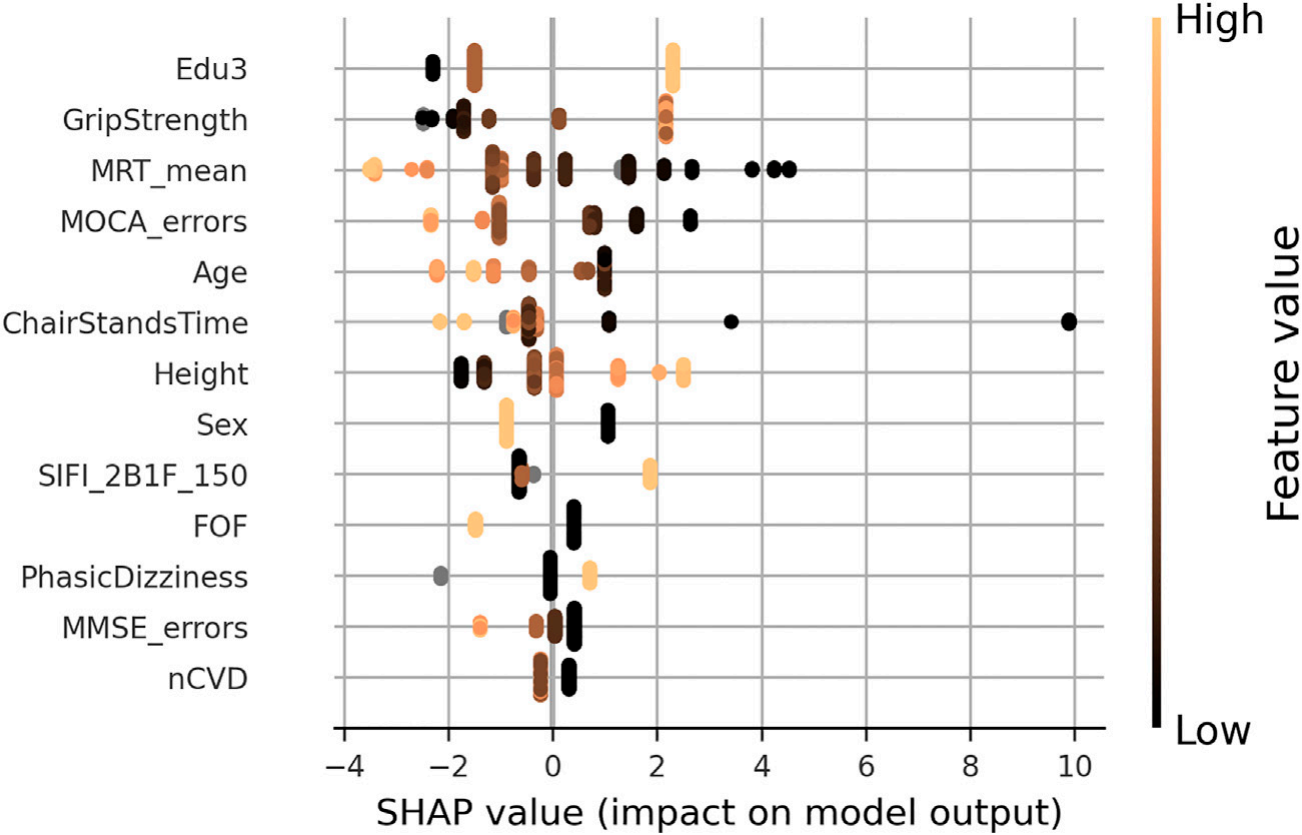
SHAP summary plot for the final gait speed reserve model. Features are ordered from top to bottom by decreasing mean absolute SHAP value. For each feature, each point represents a single sample in the test data. A sample’s x-coordinate displays the SHAP value for that sample with respect to the given feature. The colour of a sample indicates the value of the feature, with light brown being high, black low, and grey missing.

In the SHAP summary plot for the GSR model shown in Figure 11, the feature importance ranked in order of decreasing mean absolute SHAP values was level of educational attainment, grip strength, mean MRT, MOCA errors, age, chair stands time, height, sex, accuracy proportion in the sound induced flash illusion, fear of falling, orthostatic intolerance, MMSE errors, and number of cardiovascular conditions.

**FIGURE 12:**
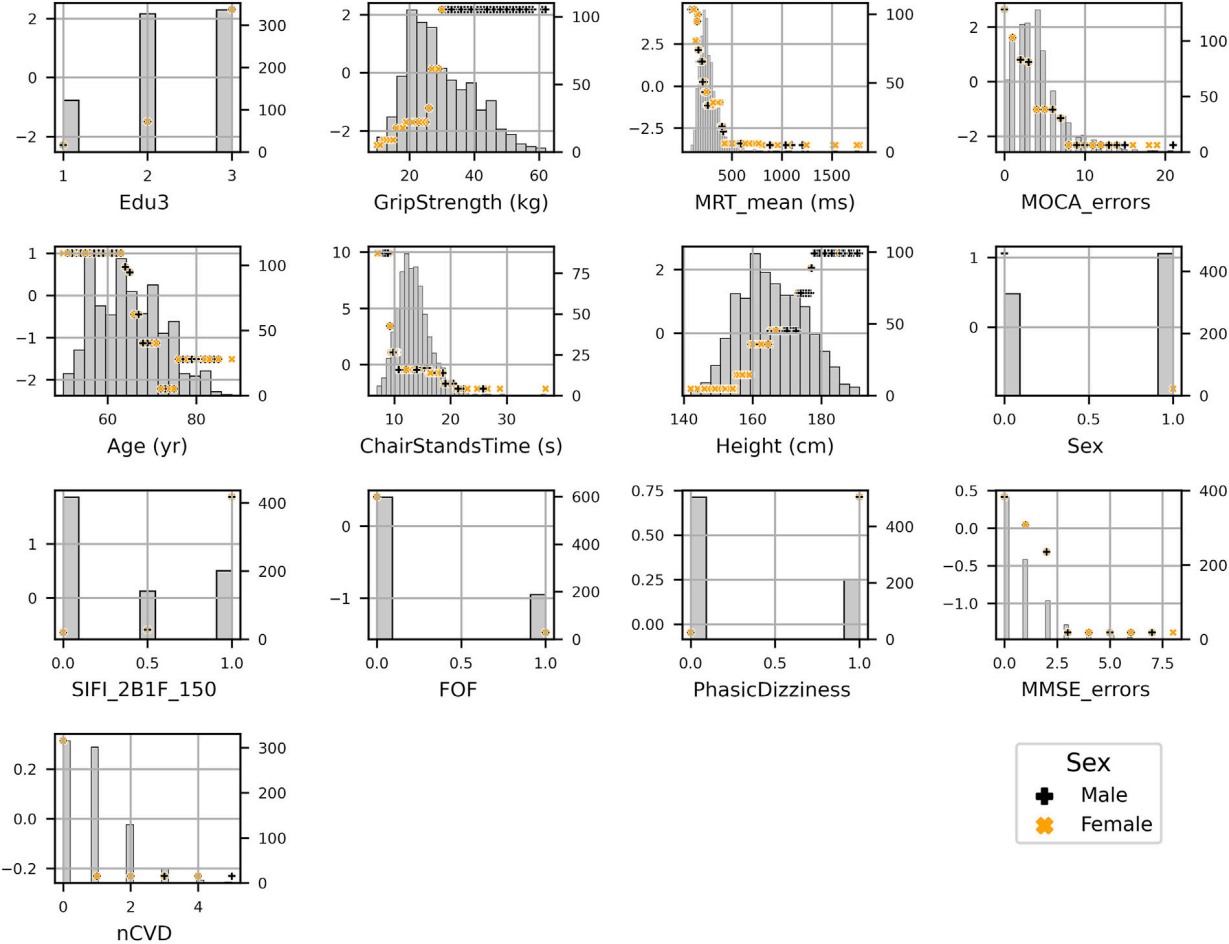
SHAP values (left *y*-axis) vs. input feature value (x- axis) with underlayed histogram (right *y*-axis shows histogram counts) for each feature in the gait speed reserve model. Points are coloured per sex: male is black “+,” and female is orange “x.”

Figure 12 shows the SHAP values versus input feature values with underlaid histogram for each feature in the GSR model. The absence of vertical spread in the SHAP vs. feature scatter plots is due to the maximum leaf nodes hyperparameter being set equal to two for the histogram gradient boosting model. This results in there being no interaction terms, since the predictions made by each tree only considered features independently (i.e., a maximum leaf node limit of two means that for a given tree only a single split is made along a single feature).

**FIGURE 13:**
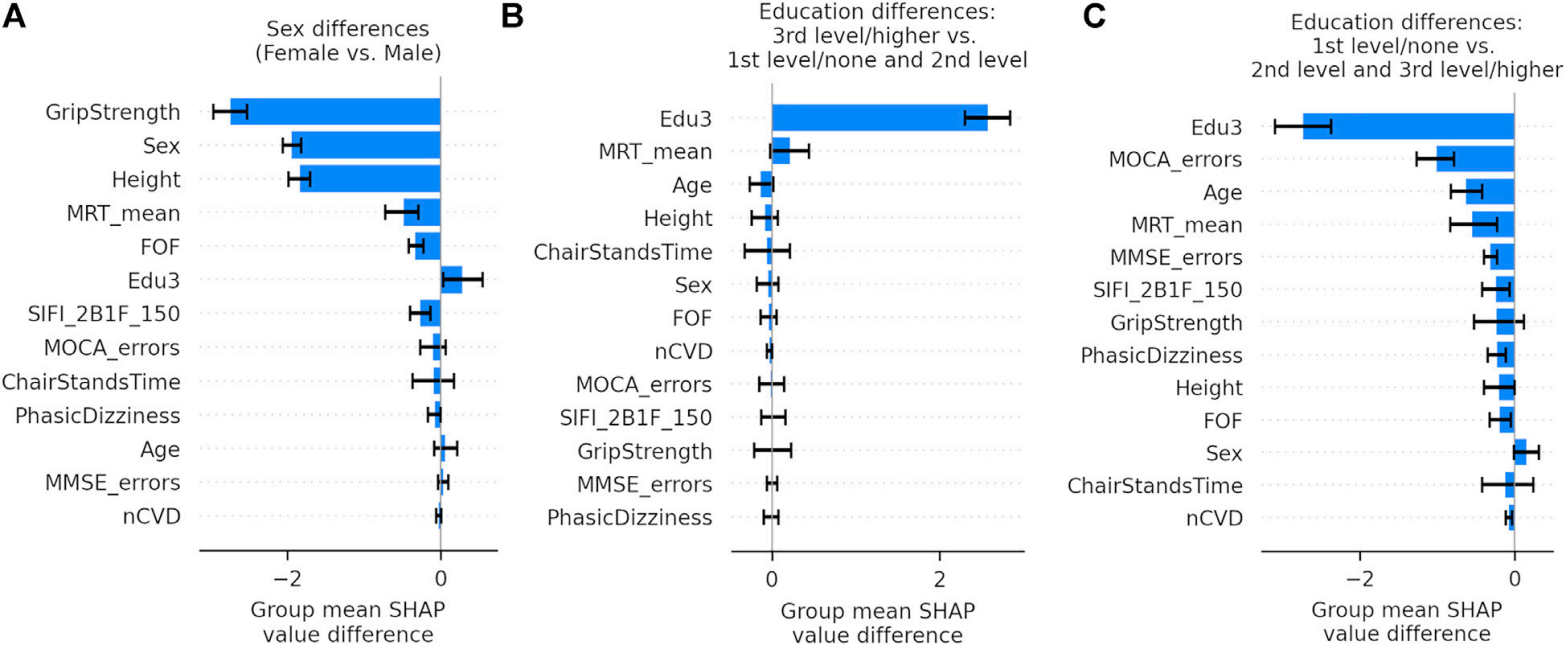
Bar graphs showing the group mean differences in SHAP values between subgroups with 95% confidence intervals for each feature in the gait speed reserve model. Panel **(A)** shows the differences in sex, Panel **(B)** shows the differences between participants with third/higher level of educational attainment and all others, and Panel **(C)** shows the differences between participants with first/no level of educational attainment and all others.

The group mean differences in SHAP values for each feature along with 95% confidence intervals can be seen in Figure 13 for (A) sex, (B) third level education vs. all others, and (C) first/no education vs. all others. For sex, the grip strength feature produced a larger difference in means than sex itself with grip strength having a less positive impact for women. Height, mean MRT, fear of falling, and SIFI accuracy were all significant, and all exhibited a negative mean impact difference. On the other hand, for education, there was a positive group mean difference for women in comparison to men. When comparing third/higher educational attainment to the rest, education itself seemed to make the only significant difference. However, when comparing primary/no educational attainment to secondary and tertiary educational attainment in Figure 13C, we observed several other significant differences other than education: MOCA errors, age, mean MRT, MMSE errors, illusion accuracy, orthostatic intolerance, fear of falling, and number of cardiovascular diseases.

### Summary of Results

**FIGURE 14:**
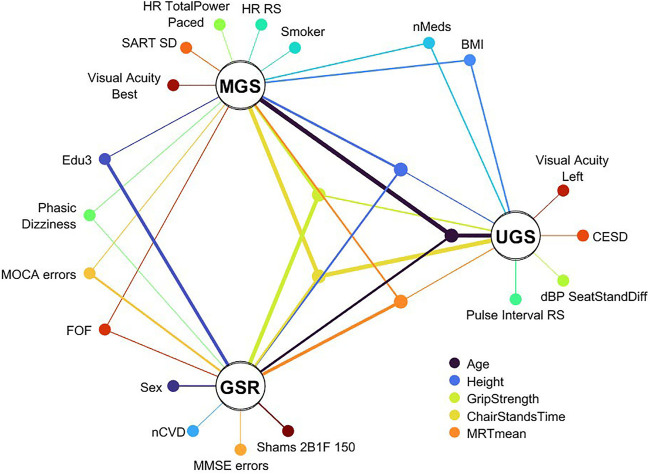
Graphical summary of features selected for the three models. Features unique to a model are shown positioned around that model’s node. Features common to all models are positioned in the central ring of nodes; the legend for those five features is located below the UGS node. The remaining nodes are for features common to two of the models. The thickness of lines connecting feature nodes to model nodes express a normalized mean absolute SHAP value of that feature in that model. The normalization is performed per model to reflect the relative importance of a feature to that specific model. The colour of the features and correspondent link depends on the subset of features: gradations of blue (from dark blue to turquoise) for socio-demographics/anthropometrics/medical history features, light green for cardiovascular features, yellow-green for physical strength features, gradations of orange and light red for cognitive and psychological domain, and dark red/brown for sensory features.

Supplementary Appendix C, Table 1 summarizes the scores and features selected for each model. The network between predictors and the three gait variables is visually summarized in Figure 14. The outcomes UGS, MGS, and GSR constitute the main nodes of the network and are represented by white circles. Smaller nodes of different colours represent distinct features and are spatially organised according the following: at the centre, there are the features that have been automatically selected in the models for all three outcomes and, therefore, are common to all the outcomes; externally to the central network, disposed on a peripheral imaginary circle, there are features that are common to just two of the three outcomes; and closely around each outcome, there are the unique feature for that particular outcome. Each link between a feature node and an outcome node represents the impact that feature has on the model output: the line thickness is proportional to the mean absolute SHAP value for that feature in the model scaled according to that model. The colour of the features and correspondent link depends on the subset of features: gradations of blue (from dark blue to turquoise) for socio-demographics/anthropometrics/medical history features, light green for cardiovascular features, yellow-green for physical strength features, gradations of orange and light red for cognitive and psychological domain, and dark red/brown for sensory features.

## Discussion

### Overall Summary of Findings

In the present study, using data from Wave 3 of TILDA, we employed a gradient boosted trees-based stepwise feature selection pipeline for the discovery of clinically relevant predictors of GSR, UGS, and MGS using a shortlist of 88 features across 5 domains. The features selected for the respective models explained MGS and UGS to a greater extent than GSR. As shown in Figure 14, there were common features but also some features unique to each of the three models.

### Model Prediction

Based on model $R_{adj}^{2}$ values, GSR (19%) was less predictable than MGS (45%) and UGS (38%). Whilst we are not aware of previous published data for comparison with our GSR prediction, a previous study by Bohannon reported linear regression *R* ^2^ values of 13% for UGS and 41% for MGS (Bohannon, 1997). Our results agree in that the MGS model yielded a larger prediction score than that of UGS but are comparably superior, especially given the fact that our *R* ^2^ unit is adjusted.

### Common Features

Across the three models, there were five common selected features: age, grip strength, chair stands time, mean motor reaction time in the choice reaction time test, and height. The top 4 features with the most impactful interactions (by mean absolute SHAP interaction value) were the same for the UGS and MGS models: age, chair stands time, grip strength, and BMI.

Our results agree with Bohannon’s previous findings that UGS and MGS decline with increasing age (Bohannon, 1997). Other authors have also shown similar findings for UGS (Samson et al., 2001; Romero-Ortuno et al., 2010; Schimpl et al., 2011). As per SHAP value vs. feature plots, increasing age was negatively associated with UGS, MGS, and GSR at ≥68, ≥68, and ≥66 years, respectively. Height is also unsurprising as a common predictor; indeed, taller people have longer legs and can achieve longer strides and higher velocity in any gait modality. Consequently, gait speed is often normalized by height (Bohannon, 1997; Kenny et al., 2013; Kasović et al., 2021).

It is also clinically plausible that higher grip strength (as a marker of upper limb strength) and shorter chair stands time (more representative of lower limb strength) were common determinants of all three performance metrics. Indeed, sarcopenia (low muscle mass and/or strength), of which both grip strength and the five chair stands test are indicative measures (Cruz-Jentoft et al., 2019), has been associated with reduced gait speed and poor functional outcomes in older people (Nishimura et al., 2017; Moreira et al., 2019; Perez-Sousa et al., 2019). In our models, slower chair stands time was associated with a decline in UGS, MGS, and GSR once time increased beyond 14.2, 13, and 10.6 s, respectively; whilst increases in UGS, MGS, and GSR began at values of 13.4, 13, and 10.6 s, respectively. Grip strength of ≤26 kg was associated with slower UGS, MGS, and GSR, whilst grip strengths of ≥35, 27, and 27 kg, respectively, were associated with faster performance. These values for grip strength, whilst interesting from an absolute point of view, have a reduced clinical significance given the large differences in grip strength between men and women. Except for height, the other features relationships to the model output appear quite homogeneous with respect to sex.

Higher mean motor reaction time in the choice reaction time test was associated with lower speed in all three models. In previous research, a shorter CRT has been associated with faster gait speed after adjusting for potential confounders and suggests that, in older adults, engaging more frequently in cognitively stimulating activities may improve neuromotor performance and mobility (Cai et al., 2020). In addition, our results resonate with previous TILDA work utilizing traditional linear statistics showing that participants in the slower MRT group (<250 ms) at Wave 1 seemed to have faster mobility decline as assessed by the timed up and go at Wave 3, 4 years later (Chintapalli and Romero-Ortuno, 2021). Interestingly, in the latter study, the MRT cutoff was set arbitrarily, but in the present study, the negative/positive impact thresholds for UGS, MGS, and GSR were 299, 231, and 229 ms, respectively. Interestingly, the less physically demanding UGS model was only negatively influenced above a relatively slower MRT threshold.

The counter-intuitive results of higher grip strength, quicker chair stands time, and quicker MRT being associated with an increase in UGS when compared to MGS may be revealing underlying determining mechanisms of both acts; MGS may be a more physically determined act than UGS and easier to improve on than UGS.

Common between UGS and MGS models were BMI and number of medications, in the clinically expected directions, i.e., obesity and number of medications had a negative impact on gait speed. As regards obesity, research has suggested that obese adults may select their walking speed to minimize pendular energy transduction, energy cost, and perceived exertion during walking (Fernández-Menéndez et al., 2019). In our UGS and MGS models, a BMI ≥29 kg/m^2^ had negative impact association. Hypothetically, it is possible that in TILDA, obese individuals equally reduced their UGS and MGS, which could possibly explain why BMI was not a feature in the GSR model. As regards the number of medications, a similar mechanism could apply. In any case, our findings are in keeping with previous research showing that drug interactions may increase the likelihood of gait speed decline amongst older adults (Naples et al., 2016). In our UGS and MGS models, more than two medications had a negative impact association. This is below the usual polypharmacy definition of 5+ medication, and the negative impact association with medications could be related to the underlying health condition rather than due to the medications themselves. Of note, visual acuity featured in both UGS (left) and MGS (best), but not in GSR, which could have a similar underlying reason (i.e., both UGS and MGS equally limited).

There were no features exclusively shared by UGS and GSR, but there were four features in the intersection of MGS and GSR: education, MOCA errors, fear of falling, and orthostatic intolerance. As regards the former two, tertiary education was associated with increased gait speed and primary and secondary levels with a decrease. Greater than three MOCA errors negatively impacted both models. Interestingly, better MOCA performance is associated with higher education (Borda et al., 2019) and places greater emphasis on frontal executive function and attention tasks than the MMSE (Wong et al., 2013). Planning for the MGS task may require greater attention and executive function than performing the UGS task (Umegaki et al., 2018), and this may explain MOCA being related to GSR and MGS. Two or more MMSE errors were associated with GSR decrease.

Analogously, orthostatic intolerance and fear of falling may selectively limit the more demanding MGS task but not the more comfortable UGS task. Orthostatic intolerance can be caused by orthostatic hypotension, which in some studies has been associated with reduced gait speed (Briggs et al., 2020). In addition, orthostatic intolerance can be a feature of vestibular disorders such as benign paroxysmal positional vertigo (BPPV) (Jeon et al., 2013), and research has suggested that the gait characteristics of BPPV can be attributed to an inadequate, cautious gait control (Schniepp et al., 2014), which may preferentially manifest in the MGS task. Fear of falling can also become stronger when facing the MGS task, compared to walking at UGS (Bueno et al., 2019).

### Unique Features

Features exclusive to UGS were depression, diastolic blood pressure drop from sitting to standing, and resting-state pulse interval. Higher levels of depressive symptoms have been associated with worse performance in specific quantitative gait variables in community-residing older adults, including lower velocity (Brandler et al., 2012). In our model, CESD negatively impacted UGS when CESD >2 points.

Similarly, TILDA work showed that slower recovery of BP after standing (systolic and/or diastolic) was independently associated with poorer gait performance (Briggs et al., 2020). On the other hand, a higher pulse interval indicates a higher heart rate variability and a more parasympathetic-driven autonomic cardiac control, which has been associated with healthier states (Abad et al., 2014) and mirrors the fact that, for the UGS model, higher pulse intervals had positive influence. In our model, a baseline pulse interval of 799 ms or less had a negative impact on UGS (this is roughly 75.1 bpm: 60 s/min/0.799 s per beat).

Exclusive to the MGS model was the standard deviation of the mean reaction time in the sustained attention to response task, smoking, the mean heart rate pre-active stand, the total power of the heart rate during paced breathing, and the root mean square of successive differences between heartbeats during paced breathing. In a previous study, community-dwelling participants who displayed poorer sustained attention walked more slowly during both single and dual gait tasks (Killane et al., 2014). In our model, standard deviation of the mean SART reaction time <157.7 ms was associated with slower MGS. Interestingly, research has shown that, in habitual smokers, smoking acutely reduces baseline levels of vagal-cardiac nerve activity and completely resets vagally mediated arterial baroreceptor-cardiac reflex responses (Niedermaier et al., 1993), which could be in keeping with heart rate and heart rate variability features being selected in this model. A baseline heart rate of 67.9 bpm or more had a negative impact on MGS in our model. Comparing this to the pulse interval of 799 ms (equivalent to 75.1 bpm) associated with the beginning of negative impact association in the UGS model, we see that in terms of an increasing heart rate, MGS begins to decline earlier than UGS.

Finally, features exclusive to GSR were accuracy proportion in the sound-induced flash illusion (two beeps and one flash with stimulus-onset asynchrony of +150 ms), sex, MMSE errors, and number of cardiovascular diseases. Male sex was associated with increased GSR, potentially because men may comparatively accelerate more than women during the MGS task. Alternatively, this may also be because the variance explained by the GSR model was relatively low and the effect of sex might disappear when additional features are selected as in other models. One or more cardiovascular diseases was negatively associated with GSR, which is in keeping with the possibility that this type of disease may limit MGS more than UGS. As noted by a previous study (Clark et al., 2013), the difference between UGS and MGS is predominantly dictated by the latter. A notable exclusive associate of GSR was the proportion of accuracy in the sound-induced flash illusion. This can be interpreted in the context that worse visual–somatosensory integration is associated with worse balance in older people (Mahoney et al., 2019) and that an increase in susceptibility to the sound-induced flash illusion during standing relative to sitting was present in fall-prone older adults (Stapleton et al., 2014).

### Strengths of the Methodology and Study

A main strength of the methodology is the use of the histogram gradient boosting regressor machine learning model that bins values for faster computation; offers native support for categorical features without the need for one-hot encoding (dummy variables); has native support for missing values not requiring removal of features/samples or imputation procedures; obviates the need to scale features as it is based on decision trees; allows for non-linear relationships, making no assumptions about underlying structure; and is capable modelling feature interactions. The native support for both categorical features and missing data, together with not needing to perform scaling, reduces the time and effort required during data pre-processing. This is especially useful in the feature selection stage of a study where many features that do not end up in the model would otherwise still have to undergo those pre-processing steps.

The use of a tree-based machine learning model such as HGBR leads to another strength in that it allowed for exploration of the input–output relationships by way of the TreeExplainer explainable machine learning method from SHAP. So far, TreeExplainer is the only SHAP method that allows for exact computation of Shapely values, which, with theoretical grounding in game theory, are used to assess the contributions of features to the model output. SHAP values allow for visualizations of input–output relationships and of the contributions of feature interactions. They can also be used to derive feature importance metrics that are built up from the contributions from each individual sample in the test data.

With the SHAP value versus feature plots, one can recognize the presence of what could be considered as “floor” and “ceiling” effects in the features. This highlights the importance of using non-linear models in this type of research, as even if the relationship observed within the “active” region of the feature is indeed linear, a linear model cannot detect the plateau regions and would instead return a model coefficient that underestimates the effect size in the “active” region. Potential clinical cutoffs and regions of interest for certain features are identifiable, as we have detailed above, making the models highly interpretable for clinicians. Beyond the technical aspects, the visualizations made possible by the explainable machine learning methods are also a strength for the more clinical reader. Having run a complex machine learning model, not only the associations captured between features and model output can be observed but also the relationships between feature interactions and the output. Cutoffs, regions of interest, clusters, and trends are all on show, which can allow for better insight and hypothesis generation.

Another strength of the study is the comparison of UGS, MGS, and GSR in terms of features selected to describe them from a range of 88 features across multiple domains. The TILDA data leveraged allowed for a relatively large sample size of 3,925 participants.

### Limitations of Methodology and Study

However, whilst these cutoffs and regions of interest may be able to inform the clinician, it is possible that they may vary between populations. In terms of the analytical sample, this only included TILDA Wave 3 participants who underwent the health centre assessment where gait speed tests were conducted. Even though at Wave 1, TILDA was designed as a nationally representative cohort of people aged 50 or more years living in Ireland (Donoghue et al., 2018), our analytical sample at Wave 3 is not population representative, and therefore, our results are not necessarily generalizable to the Irish population. Indeed, TILDA work showed that participants attending the health assessment centre were generally fitter than those having a health assessment in their homes (Kearney et al., 2011b), which means that other features may have been selected in the models should frailer people have been included in the analytical sample.

Despite having many advantages, the machine learning methodology also has limitations. The features selected need to be considered in terms of the “package” of features chosen for the final model. Furthermore, it cannot be assumed that features not chosen for a model are not also predictive of the outcome variable.

Even though measures were put in place to help reduce overfitting (cross-validation on training data used in choosing features and hyperparameters, and models evaluated on a held-out test dataset), in the absence of an external validation sample, the risk of overfitting still exists. Despite using held-out test data, the absence of an external validation test means that the generalizability of the results is unknown. The confidence intervals of the effects and associations are also not known in this work; however, application of bootstrapping methods may be used in future work to address this. A rigorous time complexity analysis was not performed, but given its stepwise nature, the computation time of the feature-selection step scales with the square of the number of features considered. The number of hyperparameter iterations and the k-fold cross-validation in place also scale up the computation time. Parallelized code could help to reduce computation time. The computation time can be reduced by the early stopping function that halts the feature selection if there is no improvement or a decline for two consecutive attempts. However, when (or if) this criteria is met depends on the data.

Furthermore, the models are dependent on the predictors that were entered. Even though the “shortlist” of predictors was quite comprehensive (i.e., 88 features across 5 domains), we may not have considered potentially relevant predictors that were either not measured or not shortlisted. In view of GSR being less predictable than UGS and MGS, it is possible that including additional features in the GSR model (perhaps personality/social/lifestyle factors) would improve the model prediction. Height-normalized gait speed could have been considered in the models, but this is not something that we wanted to consider *a priori* given the data-driven approach.

Another limitation touched on in the discussion is regarding the sex differences in grip strength and height. Height is not too much of an issue, as it is non-modifiable and is a common choice for gait speed normalization, but the thresholds observed in grip strength with respect to positive or negative deviation from the mean in UGS, MGS, or GSR are heavily distorted by sex. A sex-stratified investigation of grip strength in this context may be of clinical benefit given its modifiable nature and its high importance in all three models.

Finally, it must be made clear that despite the use of word “impact” when explaining the relationship of input features to the output, all results are associations and causal relationships cannot be assumed.

### Potential Clinical Relevance

The five features selected for all three models (age, grip strength, chair stands time, mean motor response time, and height) show common factors effecting UGS, MGS, and GSR: age, upper and lower body strength, physical reaction ability, and height.

Whilst there are similarities between the three gait speed models, the differences in features chosen for each model suggest that there are physiological differences in the nature of the three gait variables. This was also suggested in the different clinical associations between the gait speeds and clinical outcomes such as falls and faints. In the domain of psychology and cognition, UGS and MGS differ the most, with UGS being associated with depression, whilst MGS is associated with cognitive performance in the SART and MOCA tests (MOCA was also associated with GSR). Education was associated with MGS and GSR but not with UGS. Fear of falling being present in MGS and GSR but not in UGS could suggest that the fear may not be in relation to usual day-to-day activity and walking but instead towards moving out of comfort zone. The unique presence of MMSE and a sound-induced flash illusion variable in the GSR model could suggest that GSR is related to a cognitive and sensory domain. The sound-induced flash illusion test assesses multisensory integration. It may be possible that UGS is more reflective of baseline health and perhaps is more sensitive to negative health outcomes, leaning more towards the frailty end of the frailty-fitness spectrum (Romero-Ortuno and O'Shea, 2013). MGS and GSR, on the other hand, may reflect more of the fitness end of the spectrum, the ability to go beyond baseline towards better fitness and more reserve but not necessarily less frailty. A potential clinical take away from this work is that modifiable associates could be targeted for a particular gait characteristic with a view to improving the higher-level aspects of health such as frailty or fitness that is more linked to that variable. Given that each of the gait speed variables was predictive of potentially different health outcomes, this work shows avenues for ultimately targeting modifiable predictors of clinically meaningful outcomes.

## Conclusion

The selected variables explained a greater proportion of variation in MGS and UGS than GSR. There were common features to all three models (i.e., age, grip strength, chair stands time, mean motor reaction time in the choice reaction time test, and height) but also some unique features to each of them. By SHAP feature importance, the top 4 features were chair stands time, age, BMI, and number of medications to the UGS model; age, chair stands time, grip strength, and height to the MGS model; and level of educational attainment, grip strength, mean motor response time, and MOCA errors to the GSR model. Overall, findings on all three models were clinically plausible and support a network physiology approach (Bartsch et al., 2015) to the understanding of predictors of performance-based tasks. Each model contains features from multiple physiological systems and thus support the hypothesis that GSR and UGS and MGS are multisystem phenomena. By employing an explainable machine learning model, our observations may help clinicians gain new insights into the possible determinants of physiological reserve in older adults. Of the features selected, some are non-modifiable, e.g., age, sex, and height. Others, however, may be directly modifiable through changes in lifestyle, engaging in physical exercise, or cognitive stimulation (e.g., BMI, weight, smoking, education, chair stands time, grip strength, MOCA, motor response time, and SART). For some variables, it may be useful to focus on ensuring that a patient avoids reaching threshold values that are associated with a rapid decline in gait speed. Conversely, if engaging in rehabilitation, those threshold values may be the targets so as to reach a more stable situation with respect to walking speed. Having explored the predictors of GSR and found multisystem associations, further work will investigate whether GSR is a useful measure in predicting adverse health outcomes and if it can contribute to informing on overall physiological reserve.

The machine learning approach allowed for the stepwise selection of the set features that best explained a target variable in a non-parametric manner that can also capture high-order interactions. Explainable machine learning allowed for the selected models to be visualized to observe the input–output relationships and the relationship between feature interactions and the model output. Using a tree-based machine learning model enabled the use of the TreeSHAP explainable machine learning package, which uses the tree structure to be able to compute exact Shapely values in low-order polynomial time. Bootstrapping will be implemented in future iterations of the method to allow for confidence intervals to be included in the model explanation visualisations.

Future work will use these methods to explore other available gait parameters and physical markers and may include new features from other domains as additional inputs.

## Data Availability

The data analyzed in this study is subject to the following licenses/restrictions: The datasets generated during and/or analysed during the current study are not publicly available due to data protection regulations but are accessible at TILDA on reasonable request. Requests to access these datasets should be directed to https://tilda.tcd.ie/data/accessing-data/.
