# Supplementary material for: Comparison of Gait Speed Reserve, Usual Gait Speed, and Maximum Gait Speed of Adults Aged 50+ in Ireland Using Explainable Machine Learning
Source: Front Netw Physiol. 2021 Nov 5;1:754477. doi: 10.3389/fnetp.2021.754477 (PMC10013005; doi:10.3389/fnetp.2021.754477)
Supplement: Supplementary file 3 [file Table3.DOCX]

Appendix C

Table 1. Summary of scores and features selected for the usual gait speed, maximum gait speed, and gait speed reserve models. Features are ordered from top to bottom by decreasing mean absolute SHAP value.

|  | **Usual Gait Speed** | **Maximum Gait Speed** | **Gait Speed Reserve** |
| --- | --- | --- | --- |
| CV Mean $R_{\mathrm{adj}}^{2}$ (SD) | 0.377 (0.04) | 0.453 (0.04) | 0.189 (0.02) |
| Train $R_{\mathrm{adj}}^{2}$ | 0.427 | 0.545 | 0.224 |
| Test $R_{\mathrm{adj}}^{2}$ | 0.411 | 0.456 | 0.208 |
|  | ChairStandsTime | GripStrength | GripStrength |
|  | BMI | ChairStandsTime | Edu3 |
|  | GripStrength | BMI | ChairStandsTime |
|  | nMeds | Edu3 | MOCA_errors |
|  | PulseInterval_RS | MRT_mean | SIFI_2B1F_150 |
|  | MRT_mean | nMeds | FOF |
|  | Height | Height | Height |
|  | CESD | SART_SD | Age |
|  | dBP_SeatStandDiff | HR_RS | Sex |
|  | VisualAcuityLeft | FOF | PhasicDizziness |
|  |  | MOCA_errors | MMSE_errors |
|  |  | PhasicDizziness | nCVD |
|  |  | Smoker |  |
|  |  | HR_TotalPower_Paced |  |
|  |  | HR_rMSSD_Paced |  |
|  |  | VisualAcuity |  |
